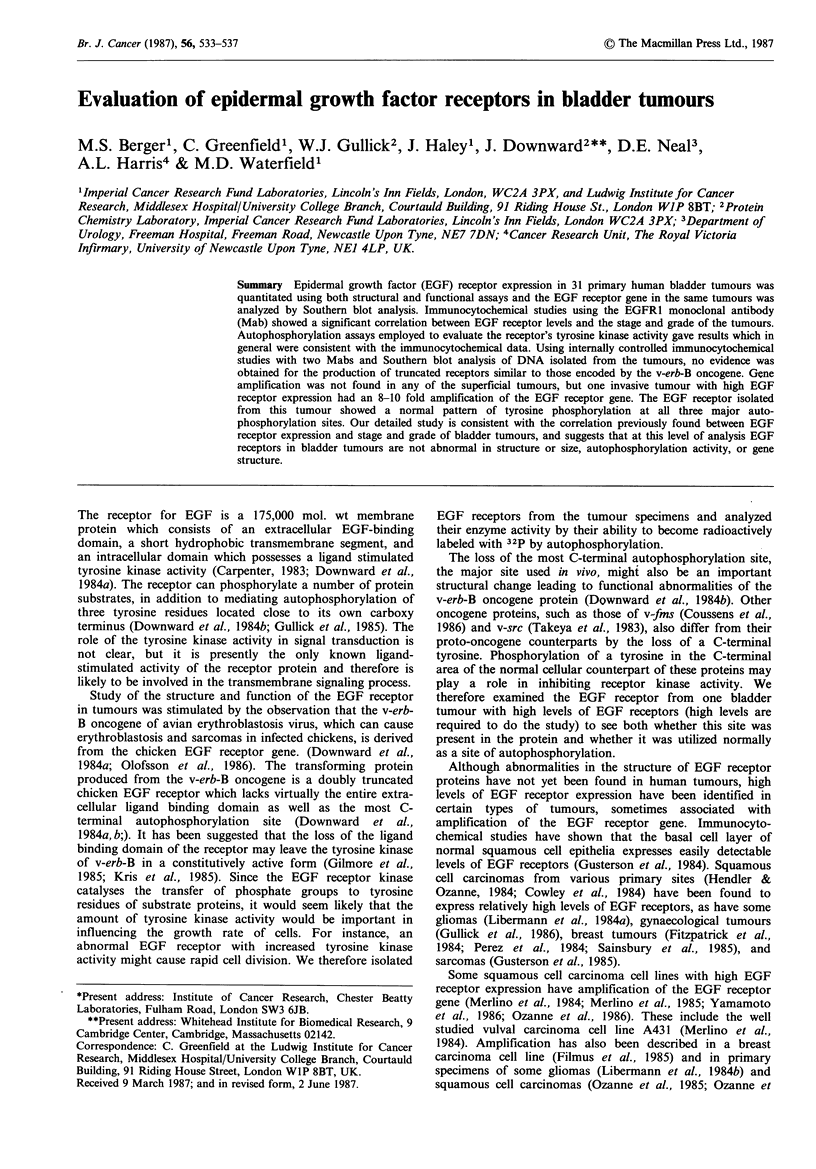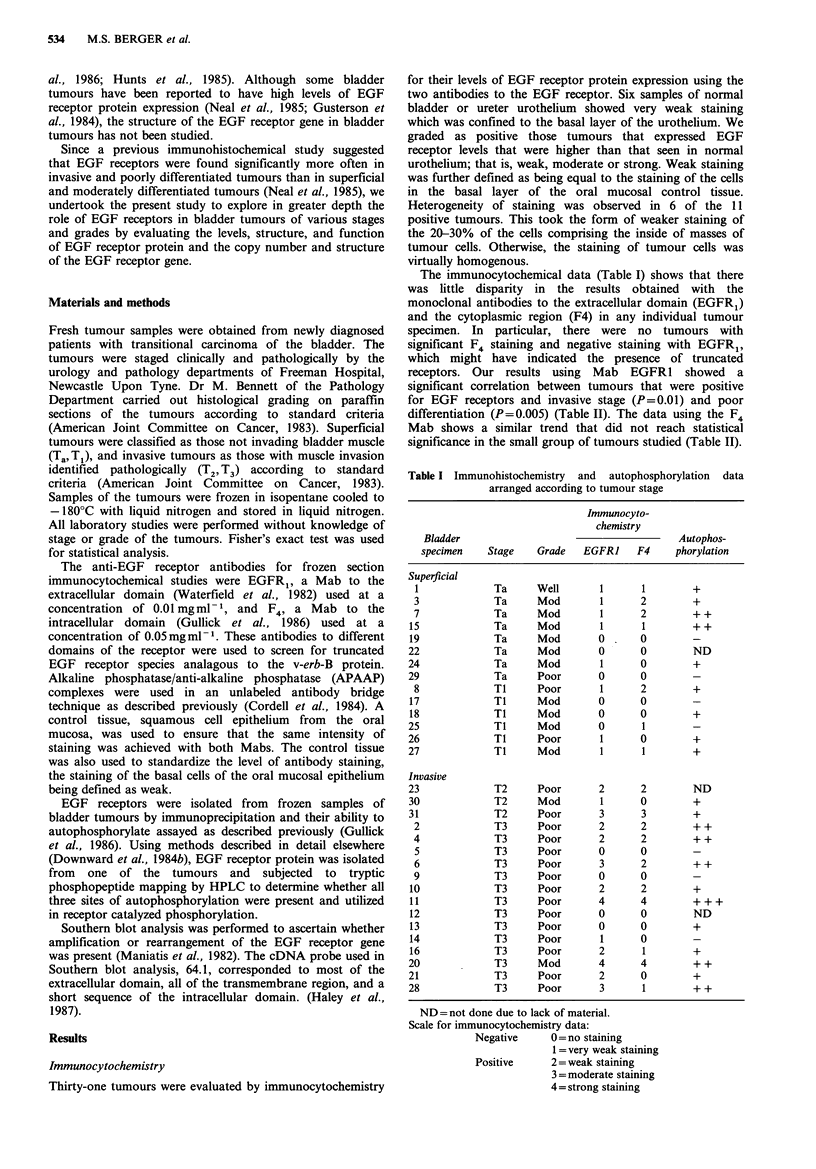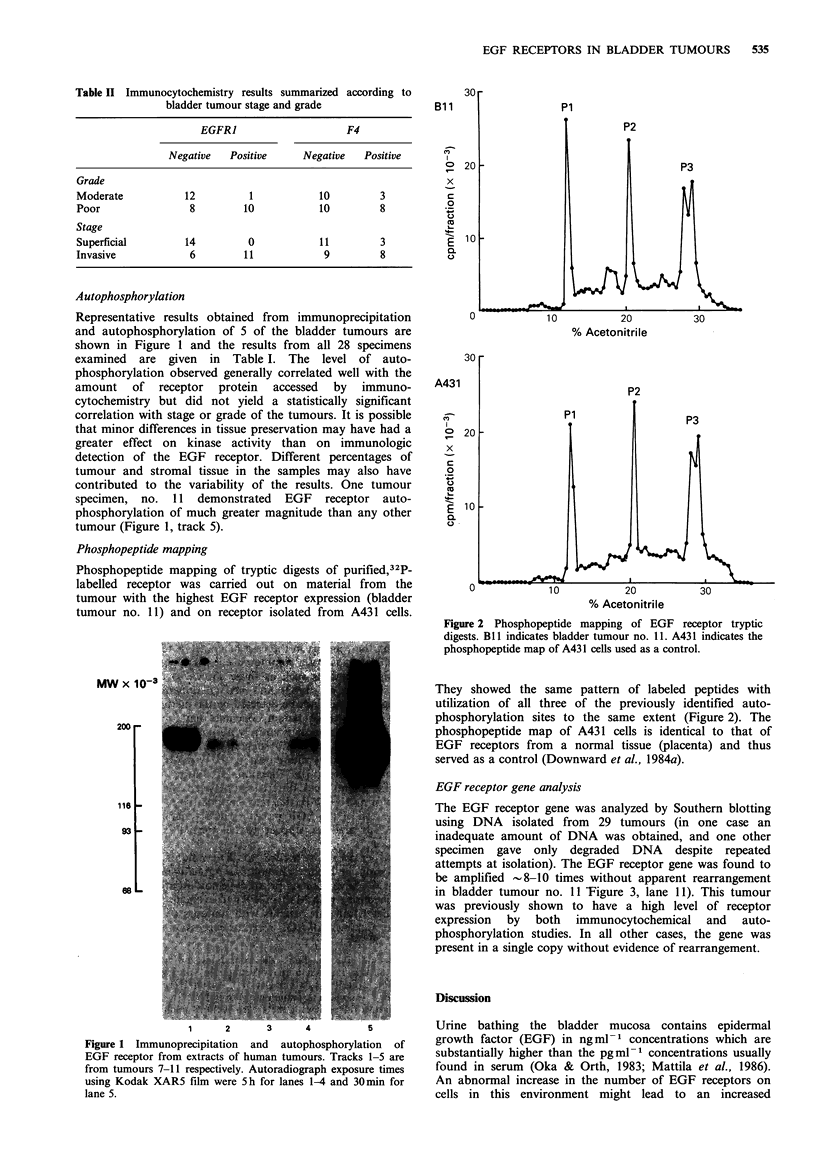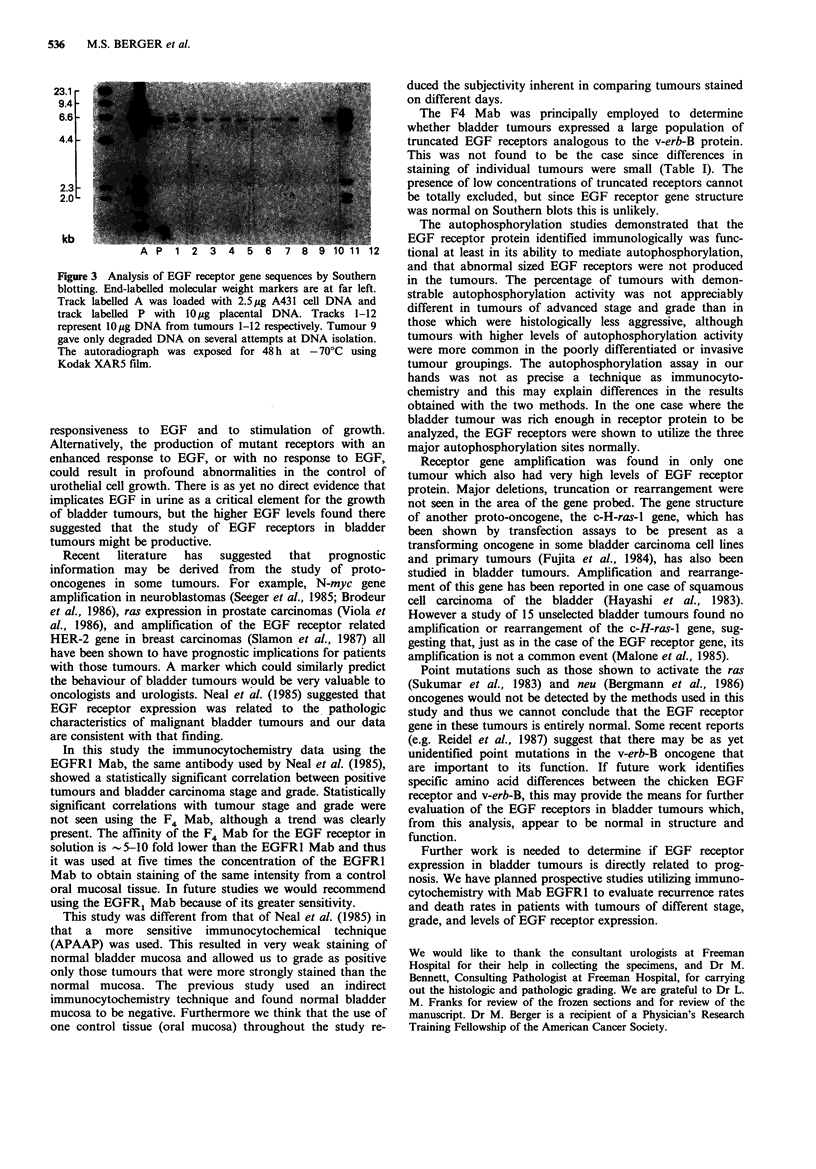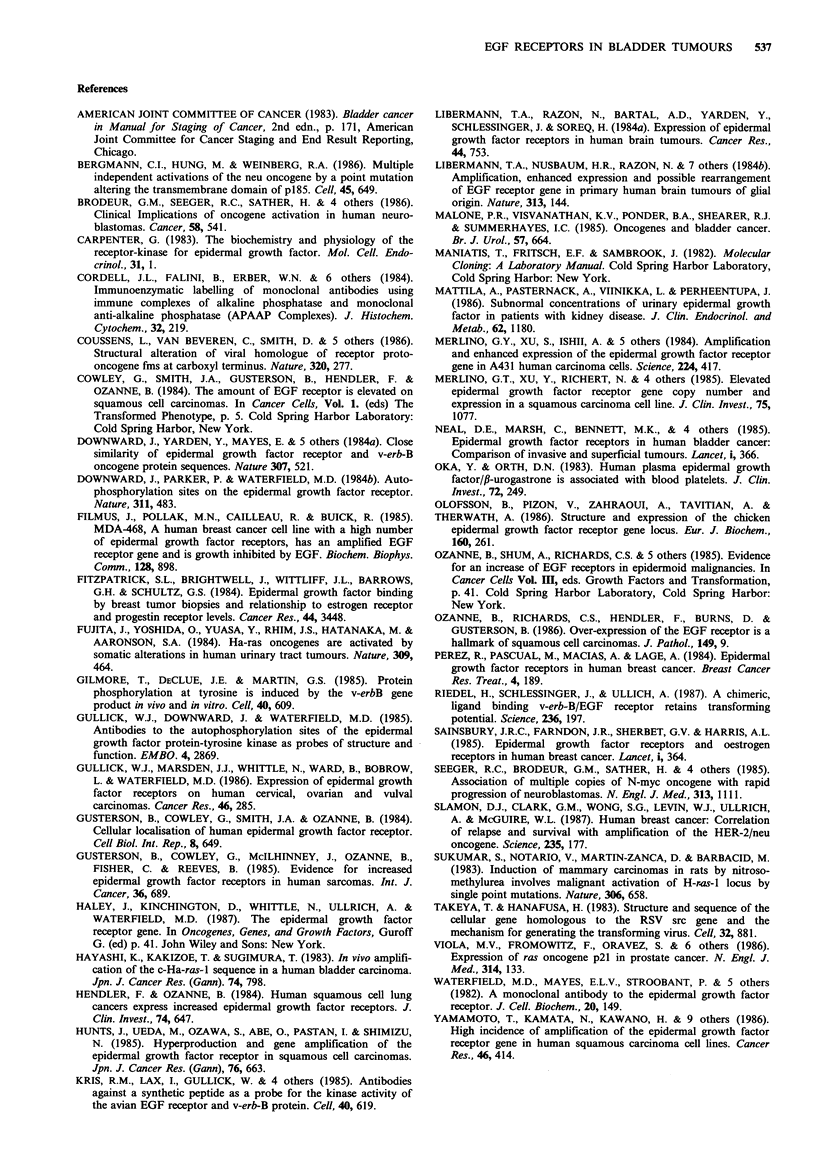# Evaluation of epidermal growth factor receptors in bladder tumours.

**DOI:** 10.1038/bjc.1987.238

**Published:** 1987-11

**Authors:** M. S. Berger, C. Greenfield, W. J. Gullick, J. Haley, J. Downward, D. E. Neal, A. L. Harris, M. D. Waterfield

**Affiliations:** Imperial Cancer Research Fund Laboratories, Lincoln's Inn Fields, London, UK.

## Abstract

**Images:**


*Br. J. Cancer* (1987), **56**, 533–537 © The Macmillan Press Ltd., 1987

### Evaluation of epidermal growth factor receptors in bladder tumours

M.S. Berger[1], C. Greenfield[1], W.J. Gullick[2], J. Haley[1], J. Downward[2]**, D.E. Neal[3], A.L. Harris[4] & M.D. Waterfield[1]

[1]*Imperial Cancer Research Fund Laboratories, Lincoln's Inn Fields, London, WC2A 3PX, and Ludwig Institute for Cancer Research, Middlesex Hospital/University College Branch, Courtauld Building, 91 Riding House St., London W1P 8BT;* [2]*Protein Chemistry Laboratory, Imperial Cancer Research Fund Laboratories, Lincoln's Inn Fields, London WC2A 3PX;* [3]*Department of Urology, Freeman Hospital, Freeman Road, Newcastle Upon Tyne, NE7 7DN;* [4]*Cancer Research Unit, The Royal Victoria Infirmary, University of Newcastle Upon Tyne, NE1 4LP, UK.*

**Summary** Epidermal growth factor (EGF) receptor expression in 31 primary human bladder tumours was quantitated using both structural and functional assays and the EGF receptor gene in the same tumours was analyzed by Southern blot analysis. Immunocytochemical studies using the EGFR1 monoclonal antibody (Mab) showed a significant correlation between EGF receptor levels and the stage and grade of the tumours. Autophosphorylation assays employed to evaluate the receptor's tyrosine kinase activity gave results which in general were consistent with the immunocytochemical data. Using internally controlled immunocytochemical studies with two Mabs and Southern blot analysis of DNA isolated from the tumours, no evidence was obtained for the production of truncated receptors similar to those encoded by the v-*erb*-B oncogene. Gene amplification was not found in any of the superficial tumours, but one invasive tumour with high EGF receptor expression had an 8–10 fold amplification of the EGF receptor gene. The EGF receptor isolated from this tumour showed a normal pattern of tyrosine phosphorylation at all three major autophosphorylation sites. Our detailed study is consistent with the correlation previously found between EGF receptor expression and stage and grade of bladder tumours, and suggests that at this level of analysis EGF receptors in bladder tumours are not abnormal in structure or size, autophosphorylation activity, or gene structure.

The receptor for EGF is a 175,000 mol. wt membrane protein which consists of an extracellular EGF-binding domain, a short hydrophobic transmembrane segment, and an intracellular domain which possesses a ligand stimulated tyrosine kinase activity (Carpenter, 1983; Downward *et al.*, 1984*a*). The receptor can phosphorylate a number of protein substrates, in addition to mediating autophosphorylation of three tyrosine residues located close to its own carboxy terminus (Downward *et al.*, 1984*b*; Gullick *et al.*, 1985). The role of the tyrosine kinase activity in signal transduction is not clear, but it is presently the only known ligand-stimulated activity of the receptor protein and therefore is likely to be involved in the transmembrane signaling process.

Study of the structure and function of the EGF receptor in tumours was stimulated by the observation that the v-*erb*-B oncogene of avian erythroblastosis virus, which can cause erythroblastosis and sarcomas in infected chickens, is derived from the chicken EGF receptor gene. (Downward *et al.*, 1984*a*; Olofsson *et al.*, 1986). The transforming protein produced from the v-*erb*-B oncogene is a doubly truncated chicken EGF receptor which lacks virtually the entire extracellular ligand binding domain as well as the most C-terminal autophosphorylation site (Downward *et al.*, 1984*a*, *b*;). It has been suggested that the loss of the ligand binding domain of the receptor may leave the tyrosine kinase of v-*erb*-B in a constitutively active form (Gilmore *et al.*, 1985; Kris *et al.*, 1985). Since the EGF receptor kinase catalyses the transfer of phosphate groups to tyrosine residues of substrate proteins, it would seem likely that the amount of tyrosine kinase activity would be important in influencing the growth rate of cells. For instance, an abnormal EGF receptor with increased tyrosine kinase activity might cause rapid cell division. We therefore isolated EGF receptors from the tumour specimens and analyzed their enzyme activity by their ability to become radioactively labeled with $^{32}$P by autophosphorylation.

The loss of the most C-terminal autophosphorylation site, the major site used *in vivo*, might also be an important structural change leading to functional abnormalities of the v-*erb*-B oncogene protein (Downward *et al.*, 1984*b*). Other oncogene proteins, such as those of v-*fms* (Coussens *et al.*, 1986) and v-*src* (Takeya *et al.*, 1983), also differ from their proto-oncogene counterparts by the loss of a C-terminal tyrosine. Phosphorylation of a tyrosine in the C-terminal area of the normal cellular counterpart of these proteins may play a role in inhibiting receptor kinase activity. We therefore examined the EGF receptor from one bladder tumour with high levels of EGF receptors (high levels are required to do the study) to see both whether this site was present in the protein and whether it was utilized normally as a site of autophosphorylation.

Although abnormalities in the structure of EGF receptor proteins have not yet been found in human tumours, high levels of EGF receptor expression have been identified in certain types of tumours, sometimes associated with amplification of the EGF receptor gene. Immunocytochemical studies have shown that the basal cell layer of normal squamous cell epithelia expresses easily detectable levels of EGF receptors (Gusterson *et al.*, 1984). Squamous cell carcinomas from various primary sites (Hendler & Ozanne, 1984; Cowley *et al.*, 1984) have been found to express relatively high levels of EGF receptors, as have some gliomas (Libermann *et al.*, 1984*a*), gynaecological tumours (Gullick *et al.*, 1986), breast tumours (Fitzpatrick *et al.*, 1984; Perez *et al.*, 1984; Sainsbury *et al.*, 1985), and sarcomas (Gusterson *et al.*, 1985).

Some squamous cell carcinoma cell lines with high EGF receptor expression have amplification of the EGF receptor gene (Merlino *et al.*, 1984; Merlino *et al.*, 1985; Yamamoto *et al.*, 1986; Ozanne *et al.*, 1986). These include the well studied vulval carcinoma cell line A431 (Merlino *et al.*, 1984). Amplification has also been described in a breast carcinoma cell line (Filmus *et al.*, 1985) and in primary specimens of some gliomas (Libermann *et al.*, 1984*b*) and squamous cell carcinomas (Ozanne *et al.*, 1985; Ozanne *et*

*Present address: Institute of Cancer Research, Chester Beatty Laboratories, Fulham Road, London SW3 6JB.

**Present address: Whitehead Institute for Biomedical Research, 9 Cambridge Center, Cambridge, Massachusetts 02142.

Correspondence: C. Greenfield at the Ludwig Institute for Cancer Research, Middlesex Hospital/University College Branch, Courtauld Building, 91 Riding House Street, London W1P 8BT, UK.

Received 9 March 1987; and in revised form, 2 June 1987.

al., 1986; Hunts et al., 1985). Although some bladder tumours have been reported to have high levels of EGF receptor protein expression (Neal et al., 1985; Gusterson et al., 1984), the structure of the EGF receptor gene in bladder tumours has not been studied.

Since a previous immunohistochemical study suggested that EGF receptors were found significantly more often in invasive and poorly differentiated tumours than in superficial and moderately differentiated tumours (Neal et al., 1985), we undertook the present study to explore in greater depth the role of EGF receptors in bladder tumours of various stages and grades by evaluating the levels, structure, and function of EGF receptor protein and the copy number and structure of the EGF receptor gene.

### Materials and methods

Fresh tumour samples were obtained from newly diagnosed patients with transitional carcinoma of the bladder. The tumours were staged clinically and pathologically by the urology and pathology departments of Freeman Hospital, Newcastle Upon Tyne. Dr M. Bennett of the Pathology Department carried out histological grading on paraffin sections of the tumours according to standard criteria (American Joint Committee on Cancer, 1983). Superficial tumours were classified as those not invading bladder muscle ($T_a, T_1$), and invasive tumours as those with muscle invasion identified pathologically ($T_2, T_3$) according to standard criteria (American Joint Committee on Cancer, 1983). Samples of the tumours were frozen in isopentane cooled to −180°C with liquid nitrogen and stored in liquid nitrogen. All laboratory studies were performed without knowledge of stage or grade of the tumours. Fisher's exact test was used for statistical analysis.

The anti-EGF receptor antibodies for frozen section immunocytochemical studies were $EGFR_1$, a Mab to the extracellular domain (Waterfield et al., 1982) used at a concentration of $0.01\,mg\,ml^{-1}$, and $F_4$, a Mab to the intracellular domain (Gullick et al., 1986) used at a concentration of $0.05\,mg\,ml^{-1}$. These antibodies to different domains of the receptor were used to screen for truncated EGF receptor species analagous to the v-*erb*-B protein. Alkaline phosphatase/anti-alkaline phosphatase (APAAP) complexes were used in an unlabeled antibody bridge technique as described previously (Cordell et al., 1984). A control tissue, squamous cell epithelium from the oral mucosa, was used to ensure that the same intensity of staining was achieved with both Mabs. The control tissue was also used to standardize the level of antibody staining, the staining of the basal cells of the oral mucosal epithelium being defined as weak.

EGF receptors were isolated from frozen samples of bladder tumours by immunoprecipitation and their ability to autophosphorylate assayed as described previously (Gullick et al., 1986). Using methods described in detail elsewhere (Downward et al., 1984b), EGF receptor protein was isolated from one of the tumours and subjected to tryptic phosphopeptide mapping by HPLC to determine whether all three sites of autophosphorylation were present and utilized in receptor catalyzed phosphorylation.

Southern blot analysis was performed to ascertain whether amplification or rearrangement of the EGF receptor gene was present (Maniatis et al., 1982). The cDNA probe used in Southern blot analysis, 64.1, corresponded to most of the extracellular domain, all of the transmembrane region, and a short sequence of the intracellular domain. (Haley et al., 1987).

### Results

### Immunocytochemistry

Thirty-one tumours were evaluated by immunocytochemistry for their levels of EGF receptor protein expression using the two antibodies to the EGF receptor. Six samples of normal bladder or ureter urothelium showed very weak staining which was confined to the basal layer of the urothelium. We graded as positive those tumours that expressed EGF receptor levels that were higher than that seen in normal urothelium; that is, weak, moderate or strong. Weak staining was further defined as being equal to the staining of the cells in the basal layer of the oral mucosal control tissue. Heterogeneity of staining was observed in 6 of the 11 positive tumours. This took the form of weaker staining of the 20–30% of the cells comprising the inside of masses of tumour cells. Otherwise, the staining of tumour cells was virtually homogenous.

The immunocytochemical data (Table I) shows that there was little disparity in the results obtained with the monoclonal antibodies to the extracellular domain ($EGFR_1$) and the cytoplasmic region (F4) in any individual tumour specimen. In particular, there were no tumours with significant $F_4$ staining and negative staining with $EGFR_1$, which might have indicated the presence of truncated receptors. Our results using Mab EGFR1 showed a significant correlation between tumours that were positive for EGF receptors and invasive stage ($P=0.01$) and poor differentiation ($P=0.005$) (Table II). The data using the $F_4$ Mab shows a similar trend that did not reach statistical significance in the small group of tumours studied (Table II).

**Table I** Immunohistochemistry and autophosphorylation data arranged according to tumour stage

| Bladder specimen | Stage | Grade | Immunocytochemistry EGFR1 | Immunocytochemistry F4 | Autophosphorylation |
|---|---|---|---|---|---|
| *Superficial* | | | | | |
| 1 | Ta | Well | 1 | 1 | + |
| 3 | Ta | Mod | 1 | 2 | + |
| 7 | Ta | Mod | 1 | 2 | ++ |
| 15 | Ta | Mod | 1 | 1 | ++ |
| 19 | Ta | Mod | 0 | 0 | − |
| 22 | Ta | Mod | 0 | 0 | ND |
| 24 | Ta | Mod | 1 | 0 | + |
| 29 | Ta | Poor | 0 | 0 | − |
| 8 | T1 | Poor | 1 | 2 | + |
| 17 | T1 | Mod | 0 | 0 | − |
| 18 | T1 | Mod | 0 | 0 | + |
| 25 | T1 | Mod | 0 | 1 | − |
| 26 | T1 | Poor | 1 | 0 | + |
| 27 | T1 | Mod | 1 | 1 | + |
| *Invasive* | | | | | |
| 23 | T2 | Poor | 2 | 2 | ND |
| 30 | T2 | Mod | 1 | 0 | + |
| 31 | T2 | Poor | 3 | 3 | + |
| 2 | T3 | Poor | 2 | 2 | ++ |
| 4 | T3 | Poor | 2 | 2 | ++ |
| 5 | T3 | Poor | 0 | 0 | − |
| 6 | T3 | Poor | 3 | 2 | ++ |
| 9 | T3 | Poor | 0 | 0 | − |
| 10 | T3 | Poor | 2 | 2 | + |
| 11 | T3 | Poor | 4 | 4 | +++ |
| 12 | T3 | Poor | 0 | 0 | ND |
| 13 | T3 | Poor | 0 | 0 | + |
| 14 | T3 | Poor | 1 | 0 | − |
| 16 | T3 | Poor | 2 | 1 | + |
| 20 | T3 | Mod | 4 | 4 | ++ |
| 21 | T3 | Poor | 2 | 0 | + |
| 28 | T3 | Poor | 3 | 1 | ++ |

ND = not done due to lack of material.
Scale for immunocytochemistry data:
Negative 0 = no staining
1 = very weak staining
Positive 2 = weak staining
3 = moderate staining
4 = strong staining

**Table II** Immunocytochemistry results summarized according to bladder tumour stage and grade

| | *EGFR1* | | *F4* | |
|---|---|---|---|---|
| | *Negative* | *Positive* | *Negative* | *Positive* |
| *Grade* | | | | |
| Moderate | 12 | 1 | 10 | 3 |
| Poor | 8 | 10 | 10 | 8 |
| *Stage* | | | | |
| Superficial | 14 | 0 | 11 | 3 |
| Invasive | 6 | 11 | 9 | 8 |

*Autophosphorylation*

Representative results obtained from immunoprecipitation and autophosphorylation of 5 of the bladder tumours are shown in Figure 1 and the results from all 28 specimens examined are given in Table I. The level of autophosphorylation observed generally correlated well with the amount of receptor protein accessed by immunocytochemistry but did not yield a statistically significant correlation with stage or grade of the tumours. It is possible that minor differences in tissue preservation may have had a greater effect on kinase activity than on immunologic detection of the EGF receptor. Different percentages of tumour and stromal tissue in the samples may also have contributed to the variability of the results. One tumour specimen, no. 11 demonstrated EGF receptor autophosphorylation of much greater magnitude than any other tumour (Figure 1, track 5).

*Phosphopeptide mapping*

Phosphopeptide mapping of tryptic digests of purified,$^{32}$P-labelled receptor was carried out on material from the tumour with the highest EGF receptor expression (bladder tumour no. 11) and on receptor isolated from A431 cells. They showed the same pattern of labeled peptides with utilization of all three of the previously identified autophosphorylation sites to the same extent (Figure 2). The phosphopeptide map of A431 cells is identical to that of EGF receptors from a normal tissue (placenta) and thus served as a control (Downward *et al.*, 1984*a*).

**Figure 1** Immunoprecipitation and autophosphorylation of EGF receptor from extracts of human tumours. Tracks 1–5 are from tumours 7–11 respectively. Autoradiograph exposure times using Kodak XAR5 film were 5 h for lanes 1–4 and 30 min for lane 5.

**Figure 2** Phosphopeptide mapping of EGF receptor tryptic digests. B11 indicates bladder tumour no. 11. A431 indicates the phosphopeptide map of A431 cells used as a control.

*EGF receptor gene analysis*

The EGF receptor gene was analyzed by Southern blotting using DNA isolated from 29 tumours (in one case an inadequate amount of DNA was obtained, and one other specimen gave only degraded DNA despite repeated attempts at isolation). The EGF receptor gene was found to be amplified ~8–10 times without apparent rearrangement in bladder tumour no. 11 Figure 3, lane 11). This tumour was previously shown to have a high level of receptor expression by both immunocytochemical and autophosphorylation studies. In all other cases, the gene was present in a single copy without evidence of rearrangement.

### Discussion

Urine bathing the bladder mucosa contains epidermal growth factor (EGF) in ng ml$^{-1}$ concentrations which are substantially higher than the pg ml$^{-1}$ concentrations usually found in serum (Oka & Orth, 1983; Mattila *et al.*, 1986). An abnormal increase in the number of EGF receptors on cells in this environment might lead to an increased

**Figure 3** Analysis of EGF receptor gene sequences by Southern blotting. End-labelled molecular weight markers are at far left. Track labelled A was loaded with 2.5 $\mu$g A431 cell DNA and track labelled P with 10 $\mu$g placental DNA. Tracks 1–12 represent 10 $\mu$g DNA from tumours 1–12 respectively. Tumour 9 gave only degraded DNA on several attempts at DNA isolation. The autoradiograph was exposed for 48 h at −70°C using Kodak XAR5 film.

responsiveness to EGF and to stimulation of growth. Alternatively, the production of mutant receptors with an enhanced response to EGF, or with no response to EGF, could result in profound abnormalities in the control of urothelial cell growth. There is as yet no direct evidence that implicates EGF in urine as a critical element for the growth of bladder tumours, but the higher EGF levels found there suggested that the study of EGF receptors in bladder tumours might be productive.

Recent literature has suggested that prognostic information may be derived from the study of proto-oncogenes in some tumours. For example, N-*myc* gene amplification in neuroblastomas (Seeger *et al.*, 1985; Brodeur *et al.*, 1986), *ras* expression in prostate carcinomas (Viola *et al.*, 1986), and amplification of the EGF receptor related HER-2 gene in breast carcinomas (Slamon *et al.*, 1987) all have been shown to have prognostic implications for patients with those tumours. A marker which could similarly predict the behaviour of bladder tumours would be very valuable to oncologists and urologists. Neal *et al.* (1985) suggested that EGF receptor expression was related to the pathologic characteristics of malignant bladder tumours and our data are consistent with that finding.

In this study the immunocytochemistry data using the EGFR1 Mab, the same antibody used by Neal *et al.* (1985), showed a statistically significant correlation between positive tumours and bladder carcinoma stage and grade. Statistically significant correlations with tumour stage and grade were not seen using the $F_4$ Mab, although a trend was clearly present. The affinity of the $F_4$ Mab for the EGF receptor in solution is ~5–10 fold lower than the EGFR1 Mab and thus it was used at five times the concentration of the EGFR1 Mab to obtain staining of the same intensity from a control oral mucosal tissue. In future studies we would recommend using the $EGFR_1$ Mab because of its greater sensitivity.

This study was different from that of Neal *et al.* (1985) in that a more sensitive immunocytochemical technique (APAAP) was used. This resulted in very weak staining of normal bladder mucosa and allowed us to grade as positive only those tumours that were more strongly stained than the normal mucosa. The previous study used an indirect immunocytochemistry technique and found normal bladder mucosa to be negative. Furthermore we think that the use of one control tissue (oral mucosa) throughout the study reduced the subjectivity inherent in comparing tumours stained on different days.

The F4 Mab was principally employed to determine whether bladder tumours expressed a large population of truncated EGF receptors analogous to the v-*erb*-B protein. This was not found to be the case since differences in staining of individual tumours were small (Table I). The presence of low concentrations of truncated receptors cannot be totally excluded, but since EGF receptor gene structure was normal on Southern blots this is unlikely.

The autophosphorylation studies demonstrated that the EGF receptor protein identified immunologically was functional at least in its ability to mediate autophosphorylation, and that abnormal sized EGF receptors were not produced in the tumours. The percentage of tumours with demonstrable autophosphorylation activity was not appreciably different in tumours of advanced stage and grade than in those which were histologically less aggressive, although tumours with higher levels of autophosphorylation activity were more common in the poorly differentiated or invasive tumour groupings. The autophosphorylation assay in our hands was not as precise a technique as immunocytochemistry and this may explain differences in the results obtained with the two methods. In the one case where the bladder tumour was rich enough in receptor protein to be analyzed, the EGF receptors were shown to utilize the three major autophosphorylation sites normally.

Receptor gene amplification was found in only one tumour which also had very high levels of EGF receptor protein. Major deletions, truncation or rearrangement were not seen in the area of the gene probed. The gene structure of another proto-oncogene, the c-H-*ras*-1 gene, which has been shown by transfection assays to be present as a transforming oncogene in some bladder carcinoma cell lines and primary tumours (Fujita *et al.*, 1984), has also been studied in bladder tumours. Amplification and rearrangement of this gene has been reported in one case of squamous cell carcinoma of the bladder (Hayashi *et al.*, 1983). However a study of 15 unselected bladder tumours found no amplification or rearrangement of the c-*H*-*ras*-1 gene, suggesting that, just as in the case of the EGF receptor gene, its amplification is not a common event (Malone *et al.*, 1985).

Point mutations such as those shown to activate the *ras* (Sukumar *et al.*, 1983) and *neu* (Bergmann *et al.*, 1986) oncogenes would not be detected by the methods used in this study and thus we cannot conclude that the EGF receptor gene in these tumours is entirely normal. Some recent reports (e.g. Reidel *et al.*, 1987) suggest that there may be as yet unidentified point mutations in the v-*erb*-B oncogene that are important to its function. If future work identifies specific amino acid differences between the chicken EGF receptor and v-*erb*-B, this may provide the means for further evaluation of the EGF receptors in bladder tumours which, from this analysis, appear to be normal in structure and function.

Further work is needed to determine if EGF receptor expression in bladder tumours is directly related to prognosis. We have planned prospective studies utilizing immunocytochemistry with Mab EGFR1 to evaluate recurrence rates and death rates in patients with tumours of different stage, grade, and levels of EGF receptor expression.

We would like to thank the consultant urologists at Freeman Hospital for their help in collecting the specimens, and Dr M. Bennett, Consulting Pathologist at Freeman Hospital, for carrying out the histologic and pathologic grading. We are grateful to Dr L. M. Franks for review of the frozen sections and for review of the manuscript. Dr M. Berger is a recipient of a Physician's Research Training Fellowship of the American Cancer Society.

### References

AMERICAN JOINT COMMITTEE OF CANCER (1983). *Bladder cancer in Manual for Staging of Cancer*, 2nd edn., p. 171, American Joint Committee for Cancer Staging and End Result Reporting, Chicago.

BERGMANN, C.I., HUNG, M. & WEINBERG, R.A. (1986). Multiple independent activations of the neu oncogene by a point mutation altering the transmembrane domain of p185. *Cell*, **45**, 649.

BRODEUR, G.M., SEEGER, R.C., SATHER, H. & 4 others (1986). Clinical Implications of oncogene activation in human neuroblastomas. *Cancer*, **58**, 541.

CARPENTER, G. (1983). The biochemistry and physiology of the receptor-kinase for epidermal growth factor. *Mol. Cell. Endocrinol.*, **31**, 1.

CORDELL, J.L., FALINI, B., ERBER, W.N. & 6 others (1984). Immunoenzymatic labelling of monoclonal antibodies using immune complexes of alkaline phosphatase and monoclonal anti-alkaline phosphatase (APAAP Complexes). *J. Histochem. Cytochem.*, **32**, 219.

COUSSENS, L., VAN BEVEREN, C., SMITH, D. & 5 others (1986). Structural alteration of viral homologue of receptor proto-oncogene fms at carboxyl terminus. *Nature*, **320**, 277.

COWLEY, G., SMITH, J.A., GUSTERSON, B., HENDLER, F. & OZANNE, B. (1984). The amount of EGF receptor is elevated on squamous cell carcinomas. In *Cancer Cells*, **Vol. 1.** (eds) The Transformed Phenotype, p. 5. Cold Spring Harbor Laboratory: Cold Spring Harbor, New York.

DOWNWARD, J., YARDEN, Y., MAYES, E. & 5 others (1984*a*). Close similarity of epidermal growth factor receptor and v-*erb*-B oncogene protein sequences. *Nature* **307**, 521.

DOWNWARD, J., PARKER, P. & WATERFIELD, M.D. (1984*b*). Autophosphorylation sites on the epidermal growth factor receptor. *Nature*, **311**, 483.

FILMUS, J., POLLAK, M.N., CAILLEAU, R. & BUICK, R. (1985). MDA-468, A human breast cancer cell line with a high number of epidermal growth factor receptors, has an amplified EGF receptor gene and is growth inhibited by EGF. *Biochem. Biophys. Comm.*, **128**, 898.

FITZPATRICK, S.L., BRIGHTWELL, J., WITTLIFF, J.L., BARROWS, G.H. & SCHULTZ, G.S. (1984). Epidermal growth factor binding by breast tumor biopsies and relationship to estrogen receptor and progestin receptor levels. *Cancer Res.*, **44**, 3448.

FUJITA, J., YOSHIDA, O., YUASA, Y., RHIM, J.S., HATANAKA, M. & AARONSON, S.A. (1984). Ha-ras oncogenes are activated by somatic alterations in human urinary tract tumours. *Nature*, **309**, 464.

GILMORE, T., DECLUE, J.E. & MARTIN, G.S. (1985). Protein phosphorylation at tyrosine is induced by the v-*erb*B gene product *in vivo* and *in vitro*. *Cell*, **40**, 609.

GULLICK, W.J., DOWNWARD, J. & WATERFIELD, M.D. (1985). Antibodies to the autophosphorylation sites of the epidermal growth factor protein-tyrosine kinase as probes of structure and function. *EMBO*. **4**, 2869.

GULLICK, W.J., MARSDEN, J.J., WHITTLE, N., WARD, B., BOBROW, L. & WATERFIELD, M.D. (1986). Expression of epidermal growth factor receptors on human cervical, ovarian and vulval carcinomas. *Cancer Res.*, **46**, 285.

GUSTERSON, B., COWLEY, G., SMITH, J.A. & OZANNE, B. (1984). Cellular localisation of human epidermal growth factor receptor. *Cell Biol. Int. Rep.*, **8**, 649.

GUSTERSON, B., COWLEY, G., McILHINNEY, J., OZANNE, B., FISHER, C. & REEVES, B. (1985). Evidence for increased epidermal growth factor receptors in human sarcomas. *Int. J. Cancer*, **36**, 689.

HALEY, J., KINCHINGTON, D., WHITTLE, N., ULLRICH, A. & WATERFIELD, M.D. (1987). The epidermal growth factor receptor gene. In *Oncogenes, Genes, and Growth Factors*, Guroff G. (ed) p. 41. John Wiley and Sons: New York.

HAYASHI, K., KAKIZOE, T. & SUGIMURA, T. (1983). *In vivo* amplification of the c-Ha-*ras*-1 sequence in a human bladder carcinoma. *Jpn. J. Cancer Res.* (*Gann*). **74**, 798.

HENDLER, F. & OZANNE, B. (1984). Human squamous cell lung cancers express increased epidermal growth factor receptors. *J. Clin. Invest.*, **74**, 647.

HUNTS, J., UEDA, M., OZAWA, S., ABE, O., PASTAN, I. & SHIMIZU, N. (1985). Hyperproduction and gene amplification of the epidermal growth factor receptor in squamous cell carcinomas. *Jpn. J. Cancer Res.* (*Gann*), **76**, 663.

KRIS, R.M., LAX, I., GULLICK, W. & 4 others (1985). Antibodies against a synthetic peptide as a probe for the kinase activity of the avian EGF receptor and v-*erb*-B protein. *Cell*, **40**, 619.

LIBERMANN, T.A., RAZON, N., BARTAL, A.D., YARDEN, Y., SCHLESSINGER, J. & SOREQ, H. (1984*a*). Expression of epidermal growth factor receptors in human brain tumours. *Cancer Res.*, **44**, 753.

LIBERMANN, T.A., NUSBAUM, H.R., RAZON, N. & 7 others (1984*b*). Amplification, enhanced expression and possible rearrangement of EGF receptor gene in primary human brain tumours of glial origin. *Nature*, **313**, 144.

MALONE, P.R., VISVANATHAN, K.V., PONDER, B.A., SHEARER, R.J. & SUMMERHAYES, I.C. (1985). Oncogenes and bladder cancer. *Br. J. Urol.*, **57**, 664.

MANIATIS, T., FRITSCH, E.F. & SAMBROOK, J. (1982). *Molecular Cloning: A Laboratory Manual.* Cold Spring Harbor Laboratory, Cold Spring Harbor: New York.

MATTILA, A., PASTERNACK, A., VIINIKKA, L. & PERHEENTUPA, J. (1986). Subnormal concentrations of urinary epidermal growth factor in patients with kidney disease. *J. Clin. Endocrinol. and Metab.*, **62**, 1180.

MERLINO, G.Y., XU, S., ISHII, A. & 5 others (1984). Amplification and enhanced expression of the epidermal growth factor receptor gene in A431 human carcinoma cells. *Science*, **224**, 417.

MERLINO, G.T., XU, Y., RICHERT, N. & 4 others (1985). Elevated epidermal growth factor receptor gene copy number and expression in a squamous carcinoma cell line. *J. Clin. Invest.*, **75**, 1077.

NEAL, D.E., MARSH, C., BENNETT, M.K., & 4 others (1985). Epidermal growth factor receptors in human bladder cancer: Comparison of invasive and superficial tumours. *Lancet*, **i**, 366.

OKA, Y. & ORTH, D.N. (1983). Human plasma epidermal growth factor/β-urogastrone is associated with blood platelets. *J. Clin. Invest.*, **72**, 249.

OLOFSSON, B., PIZON, V., ZAHRAOUI, A., TAVITIAN, A. & THERWATH, A. (1986). Structure and expression of the chicken epidermal growth factor receptor gene locus. *Eur. J. Biochem.*, **160**, 261.

OZANNE, B., SHUM, A., RICHARDS, C.S. & 5 others (1985). Evidence for an increase of EGF receptors in epidermoid malignancies. In *Cancer Cells* **Vol. III**, eds. Growth Factors and Transformation, p. 41. Cold Spring Harbor Laboratory, Cold Spring Harbor: New York.

OZANNE, B., RICHARDS, C.S., HENDLER, F., BURNS, D. & GUSTERSON, B. (1986). Over-expression of the EGF receptor is a hallmark of squamous cell carcinomas. *J. Pathol.*, **149**, 9.

PEREZ, R., PASCUAL, M., MACIAS, A. & LAGE, A. (1984). Epidermal growth factor receptors in human breast cancer. *Breast Cancer Res. Treat.*, **4**, 189.

RIEDEL, H., SCHLESSINGER, J., & ULLICH, A. (1987). A chimeric, ligand binding v-*erb*-B/EGF receptor retains transforming potential. *Science*, **236**, 197.

SAINSBURY, J.R.C., FARNDON, J.R., SHERBET, G.V. & HARRIS, A.L. (1985). Epidermal growth factor receptors and oestrogen receptors in human breast cancer. *Lancet*, **i**, 364.

SEEGER, R.C., BRODEUR, G.M., SATHER, H. & 4 others (1985). Association of multiple copies of N-myc oncogene with rapid progression of neuroblastomas. *N. Engl. J. Med.*, **313**, 1111.

SLAMON, D.J., CLARK, G.M., WONG, S.G., LEVIN, W.J., ULLRICH, A. & McGUIRE, W.L. (1987). Human breast cancer: Correlation of relapse and survival with amplification of the HER-2/neu oncogene. *Science*, **235**, 177.

SUKUMAR, S., NOTARIO, V., MARTIN-ZANCA, D. & BARBACID, M. (1983). Induction of mammary carcinomas in rats by nitroso-methylurea involves malignant activation of H-*ras*-1 locus by single point mutations. *Nature*, **306**, 658.

TAKEYA, T. & HANAFUSA, H. (1983). Structure and sequence of the cellular gene homologous to the RSV src gene and the mechanism for generating the transforming virus. *Cell*, **32**, 881.

VIOLA, M.V., FROMOWITZ, F., ORAVEZ, S. & 6 others (1986). Expression of *ras* oncogene p21 in prostate cancer. *N. Engl. J. Med.*, **314**, 133.

WATERFIELD, M.D., MAYES, E.L.V., STROOBANT, P. & 5 others (1982). A monoclonal antibody to the epidermal growth factor receptor. *J. Cell. Biochem.*, **20**, 149.

YAMAMOTO, T., KAMATA, N., KAWANO, H. & 9 others (1986). High incidence of amplification of the epidermal growth factor receptor gene in human squamous carcinoma cell lines. *Cancer Res.*, **46**, 414.